# Contraception Usage and Workforce Trends Through 2022

**DOI:** 10.1001/jamanetworkopen.2024.6044

**Published:** 2024-04-15

**Authors:** Julia Strasser, Ellen Schenk, Qian Luo, Mandar Bodas, Maria Murray, Candice Chen

**Affiliations:** 1 Fitzhugh Mullan Institute for Health Workforce Equity, Department of Health Policy and Management, Milken Institute School of Public Health, George Washington University, Washington, DC; 2 IQVIA Government Solutions, IQVIA, Falls Church, Virginia

## Abstract

This cross-sectional study uses a national data set of medical prescription claims to examine contraception service and workforce changes from January 2019 through December 2022 in the US.

## Introduction

In 2022, *Dobbs v Jackson Women’s Health Organization* dramatically changed the landscape of reproductive health, causing ripple effects that will extend beyond abortion care. Contraception may be especially vulnerable to further restrictions and barriers, both to those seeking care and those providing it.^1^ Using a national data set of medical and prescription claims, this study examines contraception service and workforce changes from January 2019 through December 2022.

## Methods

This cross-sectional study followed the Strengthening the Reporting of Observational Studies in Epidemiology (STROBE) reporting guideline. It was approved by the George Washington University institutional review board, which waived informed consent because this study uses secondary data. We used IQVIA prescription and preadjudicated medical claims to identify clinicians providing contraception services in 2019 to 2022. IQVIA captures approximately 93% of retail pharmacy prescriptions and medical claims for approximately 191 million patients. We measured 2 outcomes: (1) the monthly volume of contraceptive service visits by type and (2) the annual number and type of clinicians. Contraception methods include new prescriptions for pills, patches, and rings and visits for intrauterine devices (IUDs), implants, injectable (depo-medroxyprogesterone acetate), vasectomy, and tubal sterilization. Data analyses occurred from August 15 to October 6, 2023, and used Stata version 18.0 (StataCorp).

## Results

**Figure 1.**
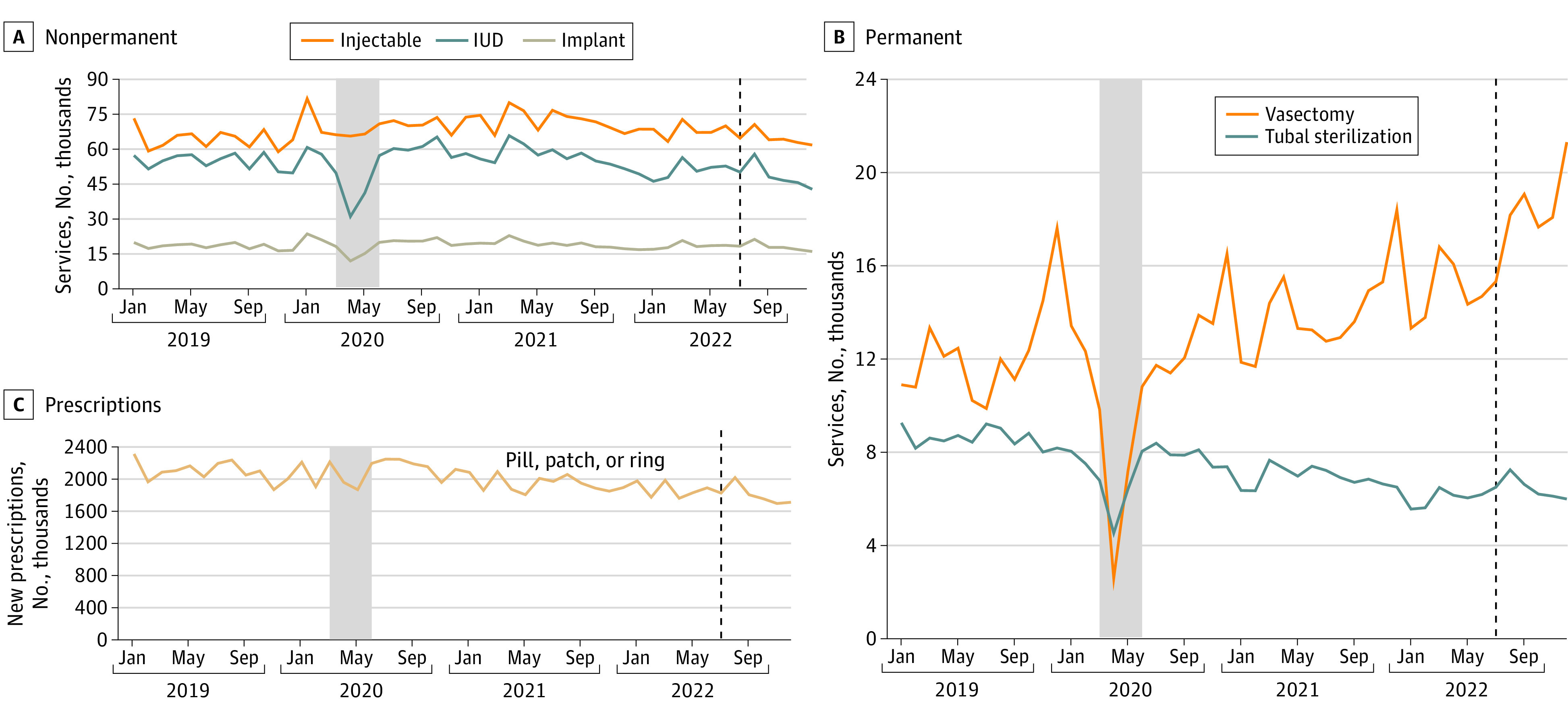
Number of Services by Contraception Method and Month, 2019 to 2022 The shaded area represents the initial months of the COVID-19 pandemic. The dotted line represents the month immediately following the *Dobbs v Jackson Women’s Health Organization* decision. Includes visits and new contraception prescriptions for all clinicians regardless of specialty. IUD indicates intrauterine device. Source: IQVIA LRx (prescription claims) and DxHx (medical and institutional claims), 2019 to 2022. Extracted June 15, 2022, and April 28, 2023.

From 2019 to 2022, 731 447 unique clinicians provided contraceptive services in our sample. Most contraception services showed steady downward trends, except for sharp declines during the early COVID-19 months and temporary increases in the month following the *Dobbs v Jackson Women’s Health Organization* decision (Figure 1).

**Figure 2.**
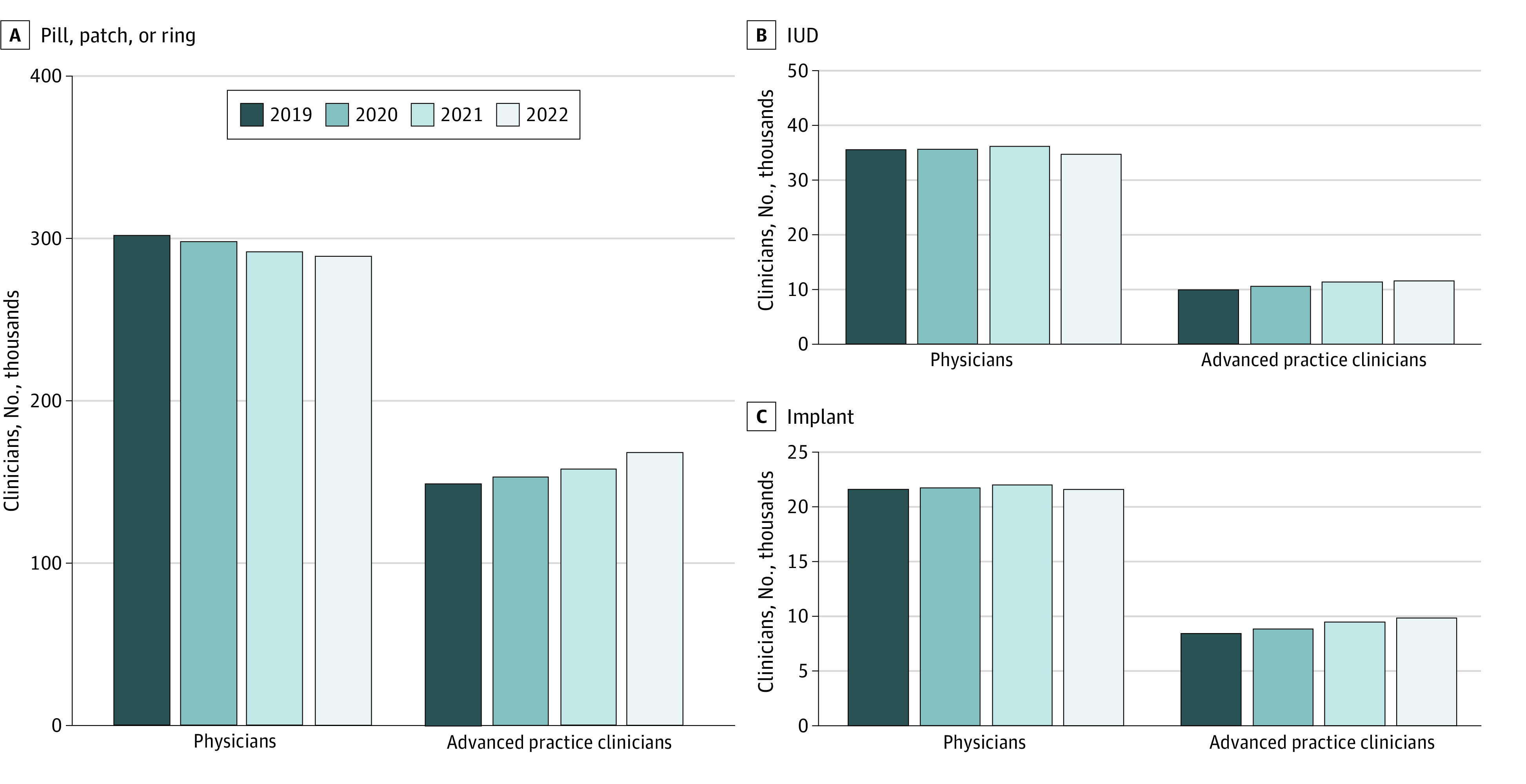
Clinicians by Profession and Contraception Method Provided, 2019 to 2022 Pill, patch, and ring clinicians prescribed at least 1 new prescription in the calendar year. Physicians include obstetricians and gynecologists, family medicine, internal medicine, pediatrics, and other physicians (eg, emergency medicine). Advanced practice clinicians include advanced practice nurses (eg, nurse practitioners, nurse midwives) and physician assistants. IUD indicates intrauterine device. Source: IQVIA LRx (prescription claims) and DxHx (medical and institutional claims), 2019 to 2022. Extracted June 15, 2022, and April 28, 2023.

The volume of IUD services decreased from 650 043 in 2019 to 591 509 in 2022, and the volume of tubal sterilization services decreased from 103 547 in 2019 to 74 537 in 2022. The volume of vasectomy services steadily increased, from 146 796 in 2019 to 198 212 in 2022. New contraceptive prescriptions (pill, patch, and/or ring) dropped from more than 25 000 000 in 2019 to less than 22 000 000 in 2022. Prescription duration increased with 44 237 865 prescriptions (68%) in 2019 limited to 1 month compared with 27 925 732 (46%) in 2022. The workforce providing contraception shifted during this period, with increases in the number of advanced practice clinicians (APCs) providing IUD, implant, and prescriptions (Figure 2) and decreases in physicians providing prescriptions.

## Discussion

While contraceptive usage increased initially in the month after *Dobbs v Jackson Women’s Health Organization*, all contraception types except vasectomy returned to overall downward trends through the end of 2022. The decreases we found in contraceptive services and the workforce providing these methods may indicate growing challenges for contraception access. News reports suggest increased interest but persistent barriers for obtaining IUDs and tubal sterilization following *Dobbs v Jackson Women’s Health Organization*,^2^ and emerging research finds some differences in post-*Dobbs v Jackson Women’s Health Organization* sterilizations by state policy climate.^3^ State-level shifts in the contraception workforce may continue, as clinicians may leave states with abortion restrictions or exit the workforce entirely.^4^

APCs are a growing segment of the contraception workforce, consistent with previous research by our team.^5^ However, the decrease in the number of physicians, coupled with state-level policies that restrict many APCs from full scope of practice, is a cause for concern.

The decreases we saw in certain contraception services may be driven by shifts in the workforce as well as patient preferences and changing markets (eg, growing presence of online pharmacies). Some of the decrease in contraception prescriptions may be due to increases in extended supply prescribing, and the growing use of vasectomy may indicate shifts in gendered approaches to reproductive decision-making.

This study has limitations. These data have limited coverage of online-only and health maintenance organization (eg, Kaiser Permanente) pharmacies. Additionally, medical claims comprehensiveness varies by individual clinician, as discussed elsewhere.^6^ As 2023 data become available, future research should continue to track changes in contraception workforce composition and access to the full range of contraceptive methods.

## Notes

Supplement.
Data Sharing Statement
