# Supplementary material for: Contraception Usage and Workforce Trends Through 2022
Source: JAMA Netw Open. 2024 Apr 15;7(4):e246044. doi: 10.1001/jamanetworkopen.2024.6044 (PMC11019391; doi:10.1001/jamanetworkopen.2024.6044)
Supplement: Supplement. — Data Sharing Statement [file jamanetwopen-e246044-s001.pdf]

Data Sharing Statement

Data
Data available: No

Additional Information
Explanation for why data not available: We used identified medical and prescription claims data which cannot be shared.
